# Spinocerebellar Ataxia Type 2: A Review and Personal Perspective

**DOI:** 10.1212/NXG.0000000000200225

**Published:** 2025-01-27

**Authors:** Stefan M. Pulst

**Affiliations:** University of Utah, Salt Lake City.

## Abstract

Spinocerebellar ataxias (SCAs) are dominantly inherited diseases that lead to neurodegeneration in the cerebellum and other parts of the nervous system. This review examines the progress that has been made in SCA2 from its initial clinical description to discovery of DNA CAG-repeat expansions in the *ATXN2* gene. *ATXN2* repeat alleles cover the range from recessive and dominant mendelian alleles to risk alleles for amyotrophic lateral sclerosis. We review studies aimed at defining the normal function of ATXN2 and mutant ATXN2 using cellular and mouse models. Progress in testing small compounds and antisense oligonucleotides in preclinical studies is described as well including our recent focus on staufen-1 (STAU1) and mRNA metabolism and control of autophagy.

## Identification of the *ATXN2* Gene

The field of degenerative cerebellar ataxias was transformed in the 1990s by genetic linkage analysis and progress in positional cloning of disease genes. This approach required ascertainment of large families, avoidance of nonallelic heterogeneity, and often the generation of novel genetic markers and physical mapping resources to identify disease-causing variants.

In retrospect, the first description of what is now known as SCA2 occurred in India in 9 families that were characterized by progressive ataxia and slow saccadic eye movements.^1^ Subsequently, a large population of individuals with a similar phenotype was discovered in eastern Cuba, especially in the province of Holguin.^2^ The high prevalence of SCA2 was attributed to a founder population in the eastern part of Cuba with 4 of every 10,000 inhabitants in Holguin diagnosed with SCA2.

Our involvement with SCA2 began in the late 1980s with the examination of a large ataxia pedigree of unknown type in New York State.^3^ We quickly excluded linkage to the SCA1 locus and began a genome-wide linkage analysis using the relatively new dinucleotide repeat genetic markers. At the same time, an international consortium headed by Susan Chamberlain at Cambridge and Georg Auburger, then at the University of Duesseldorf, mapped the SCA2 locus to the long arm of chromosome 12 between the flanking markers D12S58 and phospholipase A2 in 12 Cuban families.^4^

Independent of the international collaborative group,^4^ we established linkage to CHR12 in the family from New York State.^3^ We noticed significant anticipation of the age at disease onset from one generation to the next in this pedigree. Although unstable DNA repeat mutations and anticipation had been established at that time, there remained the question in other diseases whether the phenomenon was due to biased ascertainment in the younger generations because early-onset cases would obviously be identified first. Using flanking genetic markers, we were able to identify all at-risk individuals in this pedigree and establish that AO anticipated by 14.4 ± 7.9 years per generation, providing the first hints that SCA2 might be caused by a DNA repeat mutation.

The candidate region of the SCA2 locus in 1993 was still large, and a detailed genetic and physical map of the region was not in hand. In fact, we had to generate and map novel genetic markers using yeast artificial chromosomes (YACs) and P1 artificial chromosomes (PACs).^5,6^ This allowed us to screen for DNA repeats in PAC clones in the genetically refined region.^6^ These studies also identified one DNA CAG repeat that had 22 repeat units in controls and >35 repeats in patients with SCA2 in multiple independent families.^7^ We then screened human adult and fetal brain cDNA libraries and identified a clone with an open reading frame of 3,936 base pairs. This cDNA clone predicted an encoded protein of 1,312 amino acids with a molecular mass of 140 kD. The repeat was in exon 1 of a gene of unknown function that was subsequently designated *ATXN2* and was predicted to encode a polyglutamine (polyQ) domain. The *ATXN2* gene was large with 25 exons and showed wide tissue expression. The choice of using PACs to tile across the region was fortuitous in retrospect because the *ATXN2* repeat region was unstable in YACs.

Quite surprisingly, the SCA2 pathogenic repeat variant was identified independently and almost simultaneously by 2 other groups. The laboratory of A. Brice in Paris screened cDNA expression libraries with a monoclonal antibody that recognizes polyglutamine repeats,^8^ whereas the laboratory of S. Tsuji in Tokyo developed a technique of directly identifying and isolating expanded CAG repeats.^9^ All 3 groups identified repeat instability during meiosis and a clear inverse correlation between repeat length and age at onset.

## SCA2 Clinical Characteristics

As in many mendelian neurodegenerative diseases, identification of the SCA2 mutation allowed a reassessment and redefinition of the phenotype. While patients with SCA2 share core clinical characteristics with those with other SCAs, SCA2 is clinically distinct, when patients are considered as a group with slowed saccades being prominent. As in other SCAs, the first symptom at onset is gait ataxia. In SCA2, onset also frequently coincides with muscle cramping. Appendicular ataxia with instability of stance, dysarthria, and ocular signs including nystagmus and ocular dysmetria follow. A predominant ocular feature typical of SCA2, slow or even absent saccades, arises from involvement of brainstem neurons. Dystonia and myoclonus are also frequent in patients with SCA2, as well as neuropathy, spasticity, and frontal-executive dysfunction.^10-12^

A small number of individuals with ATXN2 mutations present with outlier phenotypes such as l-DOPA responsive parkinsonism or amyotrophic lateral sclerosis (ALS) indistinguishable from idiopathic forms of the respective diseases.^13,14^

## Architecture of *ATXN2* Genetic Variation

**Figure 1:**
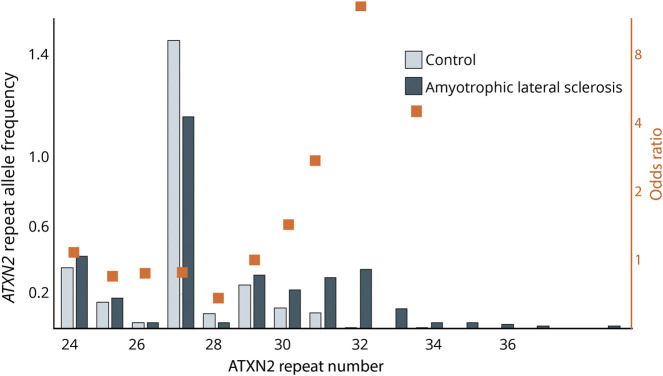
*ATXN2* CAG-Repeat Allele Frequency in Individuals With ALS and Matched Controls *ATXN2* repeat allele frequency in controls and individuals with ALS for 24 repeats or greater. Orange squares indicate allele-specific odds ratios for developing ALS (modified from Ref. 16).

*ATXN2* repeat genetic variation gives rise to mendelian alleles and risk alleles with small-to-moderate effect sizes (reviewed in Ref. 15). It is now well established that alleles with 31 and 32 repeats are recessive and associated with a cerebellar phenotype, whereas alleles with ≥33 repeats are dominant. Rarely, these alleles, especially with CAA interruptions, can cause PD or ALS phenotypes without cerebellar involvement. In 2010, the Gitler laboratory discovered a physical interaction between ATXN2 and TDP-43 leading to an analysis of *ATXN2* CAG alleles as risk factors of ALS.^14^ They found that CAG alleles with ≥27 repeats represented an increased risk of the development of ALS. Because the 27-repeat allele shows variable frequencies in different populations, unrecognized population substructure can influence risk assessment. Therefore, we examined the allele-specific risk in a meta-analysis of all extant studies at that time (Figure 1 and Ref. 16). We found that ALS risk increased significantly only for alleles with ≥31 repeats. Because alleles with 27 repeats are the third most common *ATXN2* alleles, this has important implications for genetic counseling. Alleles with 29 and 30 repeats are rare, and precise ascertainment of risk will require analysis of larger populations. Alleles with 31–33 repeats may also represent risk alleles for other neurodegenerative diseases.

## Modifiers of Age at Onset

Age at onset (AO) in polyQ-repeat disorders shows a clear inverse correlation with repeat length. In many ways, repeat variation represents a graded mutational series in that each addition of a CAG on average decreases AO by a given amount. As in other repeat diseases, *ATXN2* repeat length only explains approximately 50% of the variation in AO.

In 1999, our group was given the opportunity to attend a symposium in Havana, Cuba, with the chance to visit the Center for Research and Rehabilitation of Hereditary Ataxias (CIRAH) in Holguin. The Center comprised an impressive group of physicians, scientists, and therapists who had collected information on a significant number of patients with SCA2. We had intended to set up large-scale clinical and genetic collaboration, but politics intervened, and we were told that no DNA samples could leave Cuba for the United States. With good will from CIRAH investigators, we were finally allowed to take 100 DNA samples back to the United States for genetic analyses. Given these limitations, we decided to focus on extreme outliers of AO when corrected for *ATXN2* length because these would have the most potential to detect the influence of genetic modifiers.

With 100 DNA samples in hand, we tested the hypothesis that other SCA repeat expansion genes would influence AO. Indeed, length of the *CACNA1A* repeat associated with SCA6 influenced SCA2 AO.^17^ Of the 8 genes tested, only long-normal CAG repeats in *CACNA1A* were associated with AO after correction for *ATXN2* CAG repeat size by nonparametric tests for repeat genotypes (*p* < 0.023) and alleles (*p* < 0.04) and after correction for multiple comparisons. *CACNA1A* variation explained 5.8% of the residual variation in AO. A larger study of European and US patients with SCA2 did not confirm our results, potentially owing to unique gene × environment interactions in Cuba.^18^

In a later study, we used AO and *ATXN2* CAG repeat data to analyze the variance components by comparing AO within and in-between sibships including 129 parent-child pairs and 69 sibships.^17,19^ For the entire sample, the mutant CAG repeat allele explained 50% of variance with the remainder split between other (nonallelic) genetic variance and environmental and stochastic influences. Heritability was higher in sib-sib pairs, especially in female sib-sib pairs, than in parent-child pairs. Quite surprisingly, the AO variance components closely followed variance analyses in Venezuelan individuals with Huntington disease.^20^ These modifier studies in polyQ disorders emphasize the importance of understanding factors other than the dominant mutant allele for prognosis and therapy development.

## ATXN2 Function

Comparison of the *ATXN2* cDNA sequence with polyQ genes known in 1996 did not reveal any similarities other than the repeat domain itself. However, 2 cDNA clones were identified in the Tiger cDNA database that had high similarity. We designated this gene as ataxin-2–related protein (*A2RP*), later renamed *ATXN2L*.^7,21^ All annotated domains in ATXN2 were preserved in A2RP/ATXN2L. The same was true for mouse ATXN2^22^ lacking a polyQ tract, but with presence of Sml, SmlAD, and PABP domains and with widespread expression in brain and other tissues. Some of these domains were necessary for polyQ-induced cell death in vitro*.*^23^ The presence of domains for interaction with RNA and RNA-binding protein domains pointed to a function of ATXN2 in RNA metabolism and translation and in stress granule dynamics.

Studies by others and us confirmed this sequence-based notion and showed that ATXN2 associated with polyribosomes^24^ and localized to stress granules.^25^ A more detailed discussion of ATXN2 normal functions including implications of this locus in aging and glaucoma can be found in recent reviews.^26-28^

With a gene of largely unknown function, other than functions suggested by conserved protein domains, we began a two-pronged approach to understand the mechanisms of disease and functions of the wild-type (normal) and mutant protein. For future therapeutic approaches, it was imperative to understand whether the polyQ-expanded ATXN2 acted by loss-of-function (LoF), or dominant-negative action, or gain-of-(toxic) function (GoF).

Our initial attempt to define ATXN2 pathways used a yeast 2-hybrid interaction screen to identify proteins that bind ATXN2. Our screen identified one protein that we designated as ATXN2-binding protein 1 (A2BP1). This protein is now called RBFOX1 to highlight its similarity in structure and function to other RBFOX proteins involved in RNA splicing and other RNA functions. We and other groups subsequently identified a large number of ATXN2 interactors,^26^ some of which like parkin^23^ and TDP-43^14^ are disease-associated proteins for Parkinson disease or ALS/FTD, respectively. We found another important interactor, staufen-1 (STAU1), a decade later, this time using immunoprecipitation and mass spectrometry.^29^ ATXN2 interactions with TDP-43 and STAU1 have pointed to novel therapeutic approaches (mentioned further).

## Mouse Models

Several SCA2 mouse models have been generated including transgenic, knockin, knockout, and human bacterial artificial chromosome (BAC) lines, all summarized in a recent review.^30^

### *Atxn2* Knockout Mice

An approach toward understanding the role of ATXN2 in vivo and answering important questions regarding LoF vs GoF of the mutant allele was to inactivate *Atxn2* in the mouse and contrast phenotypes with those observed by expressing a mutant transgene. We interrupted the mouse *Atxn2* gene using homologous recombination and were surprised that, despite its strong evolutionary conservation, knockout mice were born alive, albeit with a significant segregation distortion and a significant reduction in the birth of Atxn2^−/−^ female mice.^31^ The brain including the cerebellum had normal morphology. Later studies also showed normal Purkinje cell firing in the cerebellar slice and only minimally changed cerebellar transcriptomes compared with wild-type littermates.^32^

The germline loss of *Atxn2*, however, was not completely without a macroscopic phenotype because knockout mice showed hyperactivity in the open cage setting and loss of fear learning.^33^ This was accompanied by marked obesity associated with hyperphagia.^31^ The Auburger group generated a knockout mouse independently, confirmed similar phenotypes, and found that knockout mice were also insulin-resistant.^34^ In contrast to Atxn2-deficient flies with disturbed circadian rhythm, *Atxn2*^−/−^ mice have normal levels of PER1 and PER2 immunoreactivity in the suprachiasmatic nucleus but locomotor rhythmicity is unstable.^35^

The lack of transcriptomic, neurophysiologic, or neurodegenerative changes in the cerebellum of *Atxn2* knockout mouse was consistent with a gain-of-function of polyglutamine-expanded ATXN2.

### Transgenic Animals

To model the disease state, we generated transgenic mouse models that expressed wild-type or mutant *ATXN2* targeted to Purkinje cells or using the human promoter in a BAC model. Our initial model, developed in the late 1990s, used expression of a full-length *ATXN2* cDNA under control of a modified Purkinje cell protein 2 (*Pcp2*) promoter. Although transgenic expression of wild-type ATXN2 did not result in a motor phenotype, mutant ATXN2 with a polyQ of 58 repeats resulted in impaired motor function on the accelerating rotarod.^36^ In contrast to the Pcp2-tgATXN1 animal model, Pcp2-tgATXN2[Q58] animals did not develop intranuclear aggregates. This mouse model confirmed the notion that polyQ-expanded ATXN2 had a gain-of-function effect in vivo.

Several years later, we generated additional transgenic mouse models with an earlier onset of the disease to increase usefulness for preclinical studies. We again used the *Pcp2* promoter, but this time expressed a longer polyQ domain with 127 CAG repeats resulting in an earlier onset of the motor phenotype.^37^ Detailed study of this mouse line showed that transcriptomic changes and alterations in PC firing frequency preceded cell loss by several weeks; thus, dysfunction preceded cell death providing a rational for treatment in early symptomatic stages.

**Figure 2:**
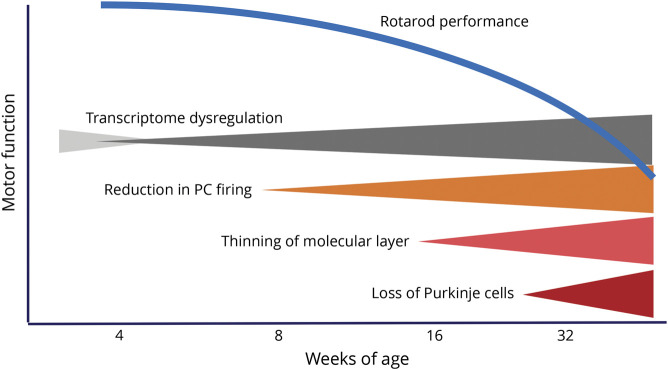
Time Course of Behavioral, Morphologic, Physiologic, and Transcriptomic Phenotypes in Pcp2-tgATXN2[Q127] Mice The curved blue line indicates progression of motor disability on the accelerating rotarod. Alterations in mRNA steady-state levels has 2 phases, an early developmental one (light gray) and a later progressive neurodegenerative one (dark gray). Note that reduction of PC firing frequency coincides with motor disability while morphologic changes occur later.

We performed a detailed analysis of progression of morphologic, biochemical, and behavioral changes in this line (Figure 2). Rotarod motor performance began to deteriorate at 8 weeks of age, but first subtle reductions in the PC number were not seen until after 12 weeks. Collaboration with the T. Otis laboratory, then at UCLA, enabled us to examine alterations of PC firing in the acute cerebellar slice. Decreases in the PC firing frequency began at 6 weeks and progressed in parallel with reduced motor performance. Most of the PCs fired regularly. Bursting cells were rare and showed no differences between wild-type and transgenic animals. Steady-state transcript changes in several PC-specific genes such as *Calb1*, *Grm1*, *Grid2*, and *Pcp2* occurred early and were progressive, with calbindin-28 K (Calb1) levels showing the first small, but significant, decreases at 4 weeks. These results emphasize that in this model of SCA2, physiologic and behavioral phenotypes precede morphologic changes by several weeks and provide a rationale for future studies examining the effects of restoration of firing frequency on motor function and prevention of future loss of PCs.

### Bacterial Artificial Chromosome

To model the widespread expression of *ATXN2* in cell types other than PCs more closely, we generated BACs that contained the entire *ATXN2* gene including all exons and introns (150 kb) with an additional 16 kb of 5′ flanking genomic sequence and 3 kb of 3′ flanking genomic sequence. We generated a line expressing ATXN2[Q72] and another one expressing ATXN2[Q22].^38^ As predicted, we found expression of ATXN2 in neurons other than PCs by laser-capture microdissected fractions of granule cells, deep cerebellar nuclei neurons, and the spinal cord. BAC-ATXN2[Q22] mice showed no morphologic or behavioral abnormalities. Despite that, it became an important resource later on for the development of antisense oligonucleotides (ASOs) targeting human *ATXN2*.

A significant number of differentially regulated genes (DEGs) were shared between the *Pcp2* and BAC models, largely related to PC-specific transcripts. This model also served as a good approach to study the effects of mt ATXN2 in the spinal cord.^39^ The BAC-ATXN2[Q22], despite lacking a phenotype, became an important in vivo model for developing ASOs and siRNAs targeting human ATXN2.

A significantly dysregulated gene in BAC-ATXN2[Q72] mice was *Rgs8* with protein levels even lower than predicted by mRNA reduction. This was due to a direct enhanced interaction between mtATXN2 and *RGS8* mRNA resulting in reduced translation, supporting the role of ATXN2 in translation regulation observed in other studies.

## Establishing Cerebellar RNA and Protein Markers for Preclinical Studies

The classic approach to characterize cerebellar degeneration has been immunohistochemistry (IHC) of PCs and their dendrites with antibodies to the calcium-binding protein calbindin 28K (CALB1). This has often focused on lobule VI because this lobule can be easily identified. CALB1 is highly expressed in PCs and involved in intracellular calcium regulation. In addition to its intensity in PCs, CALB1 staining can help visualize the molecular layer and be used to measure its thickness. The IHC procedure generally necessitates identifying a region of interest (ROI) in a tissue section and then determining intensity with a program such as ImageJ.

With an eye on developing therapeutics, we aimed to develop methods that were less operator-dependent on the choice of a ROI and that would sample an entire cerebellar hemisphere. We began by quantifying *Calb1* mRNA using reverse-transcribed PCR (qPCR) in a cerebellar hemisphere. This approach proved to be highly reproducible and faster than IHC. We found that levels of *Calb1* in the Pcp2-tgATXN2[Q127] were normal at birth, significantly downregulated at 4 weeks of age, with a progressive decline after that time point.^37^

Progressing from semiquantitative immunohistochemistry to quantitative PCR of key PC-specific genes led to the use of genome-wide transcriptional profiling. We identified the top dysregulated genes (DEGs) and identified 7 genes that had well-characterized antibodies to their respective gene products to analyze steady-state protein levels in disease progression and in response to therapeutic interventions.^32,39,40^ We found the qPCR and protein-based approaches require only 3–4 animals per experimental group because variation in technical and even in biological replicates is small compared with the 15–20 animals needed for behavioral analyses.

## Electrophysiology of SCA2 Purkinje Cells

For us as a molecular neurogenetics group, a new road to investigation of the cerebellum began with a collaboration with Tom Otis, then Chair of Neurobiology at UCLA. We started to analyze spontaneous firing of Purkinje cells in the acute cerebellar slice. By recording extracellularly, it was possible to record from a large number of PCs in the slice and provide a precise time course of decreased firing frequency. Firing frequency decreased with progression of disease and improved with gene silencing.^37,40^ Similar results were subsequently obtained in the BAC model.

The metabotropic glutamate receptor 1 (mGluR1) in PCs is essential for cerebellar function. Dysregulation of calcium homeostasis downstream of mGluR1 in genetic forms of ataxia has been hypothesized as one of the key pathologic events. We found that in Pcp2-tgATXN2[Q127] mice, calcium homeostasis in PCs was altered across a range of physiologic conditions.^41,42^ Compared with wild-type mice, mGluR1-mediated excitatory postsynaptic currents (EPSCs) at parallel fiber synapses and the associated calcium transients were greater and of longer duration. Enhanced mGluR1 function was prevented by buffering calcium at normal resting levels, while in wild-type PCs, elevated calcium increased mGluR1 EPSCs. These results led us to propose a model of a deleterious positive feedback loop that implicated elevated intracellular calcium and enhanced mGluR1 function in SCA2 mice, both contributing to PC dysfunction and loss. Further disturbance of intracellular calcium by interaction of mutant ATXN2 and ITPR1 is described as follows.

The Shakkottai laboratory explored the biophysical alterations associated with decreased PC firing in the Q127 model.^43^ They found that early in the disease process, reduced expression of large-conductance calcium-activated potassium (BK) channels and Kv3.3 voltage-gated potassium channels resulted in a deficiency of PCs to maintain repetitive spiking. Of note, mutations in *KCNC3* encoding Kv3.3 are the cause of SCA13.^44^ With PC atrophy later in the disease, spontaneous repetitive spiking at a greatly reduced frequency was restored. This was in part the result of increased activity of barium-sensitive potassium channels, likely inwardly rectifying potassium (Kir) channels. Increased Kir channel activity is the cause of a novel afterhyperpolarization that is not present in wild-type PCs.

The detailed analysis led to an important insight in that spiking changes in this SCA2 model may have different etiologies at different stages. This suggests that therapies targeted at correcting potassium channel dysfunction in degenerative ataxias may have to be tailored to the respective disease stage.

Collaboration with the A. Oro skin cancer laboratory led to interesting insights into control of PC physiology by kinases. The Oro group was exploring functions of the gene Missing in Metastasis (*MIM/MTSS1*). This gene was first identified as deleted in bladder cancer and functions in an evolutionarily conserved signaling pathway that reduces kinase activity of proteins in the Src family of nonreceptor tyrosine kinases (SFKs). The Oro group generated a conditional mutant allele in the mouse targeting the endophilin/Src-interacting domain in the final *Mtss1* exon. Surprisingly, loss of *Mtss1* resulted in cerebellar degeneration and PC death preceded by reduced PC firing in the cerebellar slice.^45,46^

This led us to examine MTSS1in Pcp2-tgATXN2[Q127] mice. Indeed, these mice had greatly reduced MTSS1 protein levels and 8-fold elevated SFK activity. Treatment with dasatinib, a clinically approved Src inhibitor, improved PC spontaneous firing in the acute cerebellar slice and delayed ataxia progression in mice lacking MTSS1 and in ATXN2[Q127] mice. Similar results were obtained with treatment of cerebellar slices by lowering activity of receptor protein-tyrosine phosphatases using peptide inhibitors.^46^

The role of Src kinases and other protein kinases, including those targeting ATXN2, has remained largely unexplored in the field of SCAs. Their analysis in vitro and in vivo could provide treatment approaches repurposing pathways and drugs well explored in the cancer field.

## Preclinical Therapies and Early-Phase Clinical Trials

Several ATXN2 interacting proteins have led to preclinical therapeutic studies. The Bezprozvanny group identified the inositol triphosphate receptor-1 (IPR1) as an interactor for mutant ATXN2 resulting in increased calcium release from intracellular stores.^47^ Targeting the functionally co-regulated ryanodine receptor with dantrolene resulted in improved phenotypes in the Pcp2-tgATNX2[Q58] mouse model.

The Orr laboratory identified cholecystokinin (CCK) as greatly reduced in SCA1 mouse models and subsequently also in ATXN2[Q127] mice.^48^ Treatment of SCA1 and SCA2 mouse models with the Cck-receptor-1 agonist A71623 improved phenotypes in both mouse models. Of interest, treatment with the Cck1R agonist normalized mTORC1 signaling, by increasing it in SCA1 models and decreasing it in SCA2 models.

In addition to pathway-focused approaches focused on channel function, calcium or autophagy signaling, we also used a pathway-agnostic therapeutic strategy by targeting the *ATXN2* gene itself. Our initial approach screened small compound libraries in HEK293 cells using an ATXN2-luciferase construct that encompassed the human ATXN2 promoter.^49^ In a dose-dependent fashion, we observed reduced *ATXN2* on treatment with proscillaridin A, 17-dimethylaminoethylamino-17-demethoxygeldanamycin (17-DMAG), and heat shock protein 990 (HSP990), known inhibitors of Na^+^/K^+^-ATPases and HSP90, respectively. Reduction of *ATP1A2* expression by RNAi in HEK293 cells resulted in reduced transcription of endogenous *ATXN2.* This was consistent with cardiac glycosides regulating *ATXN2* expression through action on Na^+^/K^+^-ATPases.

It is intriguing that studies examining different but interrelated phenotypes also identified cardiac glycosides. Two high-content compound screens looking at stress granules or ALS-related cellular abnormalities showed that exposure to cardiac glycosides normalized phenotypes.^50,51^ Unfortunately, currently available cardiac glycosides have poor permeability of the blood-brain barrier and a narrow therapeutic window. These studies, however, point to a connection between ATXN2, sodium pumps, intracellular calcium, stress granules, and ALS cellular phenotypes.

## Antisense Oligonucleotides

A chance encounter at the meeting of the Society for Neuroscience in 2012 sparked a collaboration with Ionis Pharmaceuticals aimed at developing ASOs to target *ATXN2* RNA. We opted to screen gapmer ASOs that engage RNAse H1 to degrade DNA-RNA hybrid molecules^52,53^ with the goal to reduce abundance of wild-type and mutant ATXN2 RNA.^40^ After an initial cell-based screen of ASOs, we progressed the best ASOs to screening for lowering ATXN2 levels in vivo in both our Pcp2-tgATXN2[Q127] and the BAC-ATXN2[Q72] mouse models. Because current ASOs do not cross the blood-brain barrier, we delivered ASOs by intracerebroventricular (ICV) injection. The most promising lead ASO, designated ASO7, reduced ATXN2 levels by 70–80% after a single injection and had little cytotoxicity as judged by normal postinjection levels of IBA1 (Aif1) and GFAP.

**Figure 3:**
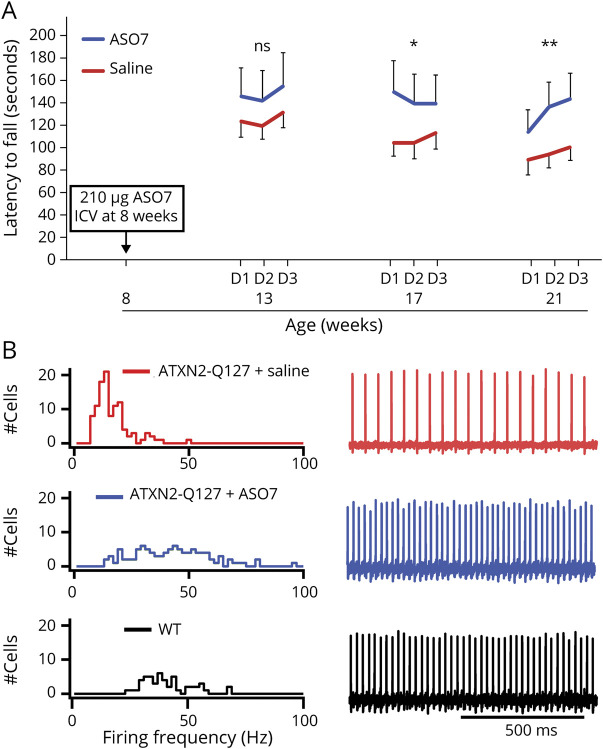
Treatment of Pcp2-tgATXN2[Q127] Mice With ASO7, a Gapmer ASO Targeting Mouse and Human *ATXN2* (A) Performance on the accelerating rotarod improves after ICV injection of ASO7 at 8 weeks. Shown are means and standard deviations for 3 daily trials per week of testing (NS = nonsignificant; *p < 0.05; **p < 0.01). (B) Normalization of PC firing frequency in the acute cerebellar slice using extracellular recordings. Left: distributions of firing frequency; right: representative firing traces for 1 second of recording.

We then conducted proof-of-principle experiments with ASO7 in both SCA2 models. We injected symptomatic mice at 8 weeks of age and tested motor behavior every 4 weeks on the accelerating rotarod. Mice were killed 13 weeks after ICV injection, and expression of mouse *Atxn2, human ATXN2, Rgs8, Pcp4, Fam107b, Homer3, Cep76,* and *Pcp2* by qPCR and Western blotting was determined. A single ASO7 treatment reduced mouse *Atxn2* and, to a greater extent, human *ATXN2* mRNA levels and improved motor performance in both SCA2 models. It also restored the mean PC firing frequency in the acute cerebellar slice to that observed in wild-type mice (Figure 3 and Refs. 40,53). Analysis of a panel of PC-specific mRNAs and proteins confirmed the treatment effect with most of them restored to near-normal levels. Of note, the treatment effect was greater at the protein than at the mRNA level, pointing potentially to a direct effect of mutant ATXN2 on translation or indirectly through elevated levels of STAU1 on RNA metabolism.

Following up on their seminal discovery that ATXN2 interacted with TDP-43 and that long-normal ATXN2 repeats were a risk factor of ALS, the Gitler laboratory examined the effects of reducing *Atxn2* in vivo. They tested this first by genetic interaction and then followed by ASO treatment.^54^

A cross of *Atxn2* knockout mice with a Thy1-hTDP-43 transgenic mouse line reduced Atxn2 by genetic interaction. Homozygous Thy1-hTDP-43 mice die within 30 days after birth. Reduction of *Atxn2* significantly extended survival in a dose-dependent fashion in that 100% genetic reduction resulted in longer survival than 50% reduction. Aggregation of TDP-43 was markedly reduced. Similar results were obtained with reduction of *Atxn2* by injecting an ASO directly after birth into the same TDP-43 overexpressing mouse line. A single ASO treatment markedly extended survival.^54^

The encouraging results from both studies prompted Ionis Pharmaceuticals to conduct a large-scale screen of ASOs resulting in a gapmer ASO (ION541/BIIB105) that was progressed to a phase 1/2 clinical trial. This ALSpire study was conducted in patients with ALS and ASO delivery by repeated lumbar intrathecal injections. The study had 2 arms for individuals with “sporadic” ALS and for those with *ATXN2* risk alleles, defined as alleles with 30–33 repeats. Over the 6-month placebo-controlled period, treatment with BIIB105 reduced ATXN2 CSF levels but did not affect clinical outcome measures. It also did not reduce plasma levels of neurofilament light chain [Ravits J, European Network for the Cure of Amyotrophic Lateral Sclerosis—20th Meeting 19 June 2024]. The study was terminated in May 2024.

## Future Developments

### Staufen

One of the intriguing ATXN2 interactors, identified by co-immunoprecipitation followed by mass spectroscopy, was staufen-1 (STAU1). Interaction of STAU1 was not different between wild-type and mutant (mt)-ATXN2, a reason why we initially considered it as not important in ATXN2 pathogenesis. However, in the presence of mt-ATXN2, STAU1 protein abundance was greatly increased in vitro and in vivo.^29,55^ Staufen was originally identified in the fly as a protein necessary for the development of mRNA gradients in early development. Its deficiency resulted in sterility.^56^ It is a multifunctional protein that binds double-stranded RNA. STAU1 protein, but not mRNA, expression was increased in SCA2 patient fibroblasts, lymphoblasts, and iPSCs. It was also overabundant in vivo in the cerebella of Pcp2-tgATXN2[Q127] and BAC-ATXN2[Q72] mice.^55,57-59^ Reducing STAU1 levels by cross with STAU1-deficient mice improved the motor phenotype of Pcp2-tgATXN2[Q127] mice.^58^ We subsequently showed that STAU1 protein levels were increased in a number of neurodegenerative conditions and associated with reduced autophagic flux owing to a direct interaction of STAU1 with the 5′-UTR of *mTOR* mRNA and its enhanced translation. Because stress granules and STAU1 are degraded by macroautophagy, enhanced translation of *mTOR* mRNA by STAU1 leads to a maladaptive feed-forward mechanism resulting in greatly reduced autophagic flux.^57^ Reducing *STAU1* by siRNA or genetic interaction can improve autophagy in several neurodegenerative in vitro and in vivo models.^58,59^ We are currently screening ASOs that target human and mouse STAU1 in models of ataxia and ALS.^60^

## Concluding Thoughts

The road from gene discovery to therapy in human patients has been more complex than anticipated. Understanding the clinical phenotype of SCA2 and the normal and pathologic functions of *ATXN2* are still ongoing almost 30 years after gene discovery. Although the first attempt at targeting *ATXN2* mRNA with ASOs has failed, the analyses of the clinical trial are not complete and efforts using approaches other than gapmer ASOs are ongoing. Targeting proteins associated with ATXN2 or modifying its abundance remains exciting opportunities for future research.

## Glossary

ALS: amyotrophic lateral sclerosis
ASO: antisense oligonucleotide
BAC: bacterial artificial chromosome
CIRAH: Center for Research and Rehabilitation of Hereditary Ataxias
IHC: immunohistochemistry
LoF: loss-of-function
PAC: P1 artificial chromosome
SCA: spinocerebellar ataxia
YAC: yeast artificial chromosome
